# Abstracts and Gleanings

**Published:** 1887-09

**Authors:** 


### ABSTRACTS AND GLEANINGS.

**Brain Surgery.**—The *British Medical Journal* says: "So that henceforwards there shall be no chyrurgeon, however ignorant in the performance of his art, which * * * may not perform this operation without any danger or fear of danger of touching the *dura mater*, the hurting whereof puts the life in jeopardy."

Thus speaks Ambroise Pare in the middle of the sixteenth century, fully aware that the opening of the dura mater in a septic wound was the most frequent cause of death in compound head injuries, and later, in the next century, Percival Pott, whose liberal use of the trephine subsequently evoked the abusive criticism of John Bell, realized the danger of following up removal of the bone by incision of the dura mater. The semi-religious respect with which the peritoneum was regarded up till thirty years ago finds its exact parallel in the dread of operative interference with the arachnoid membrane. A disputation similar to that which hindered the reform of stereotyped notions concerning the peritoneum seems likely to be imitated at this era of the birth of brain surgery proper, if certain views, which have recently found expression, are reiterated. There is, however, one fundamental difference between the introduction of surgery in the treatment of abdominal and cerebral disease respectively. In the former the mortality was at first high, in the latter it is low. The statistics of the splendid results obtained by Sir Spencer Wells in the first series of abdominal cases are in everyone's hands. Of recent cerebral operations we must understand that Mr. Victor Horsley has operated in ten cases with one death, and that from shock (the cases including four tumors of the brain); that Dr. MacEwen has had three successful cases and no deaths; Dr. Hahn, of Berlin, one successful case and no death. These, with the pioneering operation (unfortunately fatal) of Mr. Godlee; a successful Italian case, recently noted in our columns, and a case by Mr. Bennett May, show that we already possess a series of fifteen successes and three deaths (two of the latter being due to shock, the operation having been postponed too long). But we would draw attention to the fact that in this, as in every new point of departure in medical science, stress is to be laid, not on statistics of a few early cases, but on the facts of each, individually. Further proof of the value of surgical interference in cerebral disease is evidenced by the two very gratifying cases lately published in our columns; namely, one by Dr. Gowers and Mr. Barker, on December 11th, 1886, and one by Dr. Greenfield and Mr. Caird, in the *Journal* of February 12th. In reviewing the circumstances which led to the performance of the operation in these cases, it is to be noted, in the first place, that in both instances the prognosis was inevitably fatal, a state of things which, in spite of high authority, we venture to say always justifies any line of surgical treatment which is not in itself very dangerous to life. Probably every case of deliberate operation upon the brain that has been or will be performed for some time to come, has been or will be undertaken with this circumstance standing first among the factors of justification. If only one case, therefore, recovers with life, it is clear that the surgical treatment is sound, since all other was completely inadequate. Judging from slight indications, such as those to which reference is made above, the profession requires at the hands of those who are practicing in this new direction very full accounts of the cases thus relieved, and, above all, exact information on that point with which this article is begun, and which commends itself most especially to the mind of the public, namely, the guarantee that the treatment is not in itself dangerous.

The assurance of this, so far as deliberate operation on the previously uninjured head goes, is sufficiently easy, since it means nothing more than the thorough observance of certain precautions, mainly antiseptic principles. It is, however, a different question when we consider such cases as those wh ch form the subject of this article. In these instances, as a rule, the contents of the cerebral abscess are foul; and though the subdural space has to be penetrated to allow of the escape of the pus, septic infection of the meninges, which, as we have seen, has been known for centuries to be the cause of death in traumatic lesions, is, in these instances, conspicuously absent. The explanation of this is simple. As noted by Dr. Caird during his operation, simple meningitis seals the arachnoidal surface of the brain to that of the dura mater, just as in the same way simple peritonitis shuts off visceral abscesses, which, by bursting into the abdominal cavity, would infallibly cause death. The infective (septic) pus liberated from the cerebrum by the incision, and already flowing out rapidly in the direction of least resistance, is thus prevented from spreading beneath the dura mater. Fortunately, too, the intracranial pressure lends valuable aid in the obliteration of the subdural space. The actual surgical position, therefore, of this operation of trephining for cerebral abscess, is exactly that of operating for any other visceral suppuration, and may be undertaken with as little, if not less, hesitation.

The operation being thus so justifiable as to be called for, the next problem is the accurate application of such treatment; in other words, the diagnosis. We suppose that the reflection of most who read the reports of these two brilliant cases will be the somewhat discouraging one that the diagnosis in each was so uncertain as to afford little guide for future action. It is, of course, very obvious that, since the cerebral symptoms due to irritative lesion and destruction of certain portions of the brain—in these two instances the temporo-sphenoidal lobe—are masked in such cases by secondary symptoms due to pressure, etc., the diagnosis must remain somewhat obscure, even when cerebral physiology shall have discovered the functions of all parts of the brain. Meanwhile, however, it is obvious that, with the wealth of clinical research existing among the profession, a great stride forwards might be taken if all cases of cerebral abscess were most fully recorded, and if a thorough revision and comparison of the reports thus obtained were made in summary of the facts. Indeed, the minutest symptomatic detail must not be allowed to escape observation; as, for instance. the onset, spread, and character of the optic neuritis, as compared with that arising from other causes, etc. The differential diagnosis of cerebral abscess, which is a surgical emergency, from those cases which recover with or without the aid of drugs, is a difficult question, the answer to which would be honorable to the finder and valuable to humanity.—*Quarterly Compendium of Medical Science.*

**A Speedy and Sometimes Successful Method of Treating Hay Fever.**—Sir Andrew Clark, in an address delivered before the West London Medico-Chirurgical Society, describes as follows a method of procedure which he has employed with considerable success in the treatment of hay fever. For this treatment there are required a common laryngeal brush and a carbolic mixture, the latter being composed of glycerin of carbolic acid, one ounce; hydrochlorate of quinine, one drachm, and one thousandth part of perchloride of mercury, heat being required to dissolve the quinine. If there is much mucous in the nostrils, cleanse them by means of a douche of warm water containing boroglyceride, in the proportion of an ounce to the pint. Dip the laryngeal brush in the carbolic acid mixture, and see that the brush is full but not overflowing. Place the left hand on the left side of the forehead, and the thumb on the tip of the nose, with the shank of the brush between the thumb and two forefingers of the right hand, and the brush itself directed upwards, push it gently but firmly into one of the nostrils, carry it as high as you can without inflicting injury, move it about so as to bring the mixture in contact with as much as possible of the interior of the upper part of the nostril, and then withdraw it. With another brush filled with the carbolic acid mixture, or with the same brush washed, dried, and replenished, you complete in the manner following the two operations required for each nostril. Having the left hand in the position already described, and the right hand holding the laryngeal brush, with the hair pencil directed forward from the body of the operator, push the brush along the floor of the nostril into the pharynx, and after insuring free contact with the adjacent parts, withdraw it. If during the operation the brush is over-full, some of the carbolic mixture will fall into the throat and excite coughing or some other discomfort. When you have thus finished the treatment of one nostril, and carefully removed any of the carbolic acid mixture which may have been spilt upon the nose or lips, you will proceed to treat the second nostril in exactly the same manner as you have dealt with the first. During the performance of these manœuvres great assistance will be obtained from the left hand of the operator being placed over the left side of the forehead and face of the patient. With this hand the operator can adjust the patient's head to the various movements of the laryngeal brush, and with the same hand placed on the tip of the patient's nose the opening of the patient's nostril can be adjusted to a convenient size and shape. When the local effects of a paroxysm are severe, and have extended to the back part of the soft palate, it will be desirable to introduce through the mouth into the pharynx the laryngeal brush moderately filled with the carbolic acid mixture, and there, by a manœuvre easily acquired and practiced, to brush the posterior surface of the soft palate and the adjacent parts. The immediate effects of these manœuvres differ in different persons, and in the same person at different times. In all cases the effects are more or less disagreeable, and last from half an hour to half a day. Sometimes a little blood-stained mucous is discharged from the nose and throat; sometimes there is a slight frontal headache; sometimes there is a trivial cough, and occasionally you will have developed all the local phenomena of a paroxysm of hay fever.

When advising a patient with hay fever to submit to this plan of treatment for its relief, it is expedient to warn him beforehand of the disagreeable effects which sometimes follow the application of the carbolic mixture, and to assure him that they are both brief in duration and devoid of danger. When this warning is withheld, some patients will grossly exaggerate their sufferings, ascribe all sorts of injurious consequences to the application, and cover the physician with undeserved reproaches. Sometimes a single application of the carbolic acid mixture is sufficient to prevent for a whole season the return of the hay fever paroxysm, and four times within the writer's own knowledge it has never reappeared. Usually two or three applications are necessary to insure a full chance of success. The length of the interval between the applications must be determined by the character of the immediate effects. If these are mild, the application may be renewed on alternate days; but if severe, at least three days should elapse between succeeding applications.—*The Lancet, June 11, 1887.*

**Lactation and Electricity.**—Dr. Aubert (*Rev. de Therap.; Lond. M. Rec.*, May,) gives the case of a woman who had nursed her child for eleven months, when an attack of pneumonia interrupted this method of feeding. Two days later, when the infant was well enough to recommence, the secretion had completely ceased. This state of things persisted for a fortnight, during which the child, who refused other food, rapidly lost weight. It occurred to Dr. Aubert to try the effect of local faradization. The electrodes, well saturated with moisture, were placed right and left of each breast, and the current was graduated so as not to cause contraction of the pectoral muscles nor pain. After the first *seance*, which lasted twenty minutes, the right breast had evidently increased in size. The second day a few drops of milky fluid exuded, and on the third day a spontaneous flow took place in both breasts.

A second observation is reported by Dr. Becquerèl in a woman of twenty, in whom the lacteal secretion had been arrested in consequence of reiterated and intense mental emotion. After eight days' suppression three *seances* of twenty minutes' duration sufficed to re establish the secretion, which then continued in a normal way. The same result was obtained quite unexpected in a woman, seven months after her confinement, where the arrest of the secretion had been immediate.

M. Pierron has treated a number of cases by this method, and always with complete success. The mode of operating consists in placing the positive electrode, having the form of a spherical cup, over the nipple, while the negative electrode, terminating in a ball, is placed below the breast. In this manner the orifice of the gland is the first to be excited. Afterwards, the positive electrode is moved over the entire surface of the gland, while at the same time the negative electrode is displaced in such a way that the former will converge towards the latter. The current should not be strong enough to excite pain. Each application should last ten minutes, and be renewed every twenty-four hours, until milk is obtained, which rarely fails to appear after the fourth application. M. Pierron thinks that the application of electricity in this manner would produce lacteal secretion even in virgins.—*Quarterly Compendium of Medical Science.*

**A Novel and Painless Corn Salve,** according to *Magazine of Pharmacy*, consists of the following ointment, to be applied strictly according to directions:

| | | |
|---|---|---|
| ℞. | Acid salicylic | ℥ jss, |
| | Lanoline (ol. lanæ) | ℥ ijss, |
| | Creasote | ʒ ij, |
| | Petrolatum | ʒ j, |
| | White wax | ʒ j, |
| | Cocaine hydrochlor | grs. x. |
| | Alcohol sufficient. | |

Mix the two first named ingredients until perfectly smooth and homogeneous. Dissolve the cocaine in the smallest possible quantity of warm spirit, and add the solution to the creasote, petrolatum and wax (the last being previously heated until quite soft), stirring these together in a mortar until cooled to the temperature of the air, then add the first mixture, and thoroughly mix the whole. If the consistence of the finished product be not sufficiently firm when quite cold, add a little more of the wax.

This ointment may be applied directly to the affected part on pieces of lint. If the wart or corn (which may be first slightly pared with advantage) rises to a considerable height above the skin-level, it should be surrounded by a little wall or ring of hard wax (or, better, of felt or leather soaked with melted wax, and thus stiffened), the edges of which are built up a little beyond the top of the corn. In this way a kind of "well" is formed, in which a larger quantity of the discutient ointment can be deposited than is otherwise practicable. This "well" should be covered with a disc of oiled silk, gutta-percha tissue, diachylon plaster, or in any other convenient way. The ointment, of course, needs to be frequently renewed, but a very few days of such treatment will be sufficient to disorganize a portion of the corn, which is dislodged with a blunt knife, and the process repeated until there is no further work of this kind to be done. Instead of this expedient the corn plaster would no doubt answer.

The cocaine may be omitted, if desired, in all cases except those where there is much natural inflammation, or a chronic tenderness of the skin.

To relieve soreness preparatory to treatment, the application of an ointment consisting of cerate, subacetate of lead 2 ozs., oleate zinc and lanoline each 1 oz., and oil eucalyptus 8 m., is highly recommended.—*Quarterly Compendium of Medical Science.*

**A New Operation for Hernia.**—Dr. A. C. Bernays, in a preliminary communication to the *St. Louis Medical and Surgical Journal*, claims to have discovered a simple surgical operation, which is devoid of danger, and will enable any practitioner to quickly reduce strangulated hernias, whether they be oblique, or direct-inguinal, or femoral:

"The operation consists simply in the subcutaneous tenotomy of the crural arch, in other words, in cutting through Poupart's ligament. The incision is to be made about the middle of the ligament in an upward direction, and need not cut anything but the ligament itself. The method which I recommend is as follows: A small incision one-eighth of an inch in length is made through the skin only, in a vertical direction, exactly in the inguinal fold. Next, a probe-pointed knife is pushed through the subcutaneous fatty tissues under Poupart's ligament, which is found tense, and by pressing the ligament against the edge of the knife from without with the left hand, while the right hand holds the tenotome, the ligament must be cut. The knife is withdrawn as soon as the resisting ligament is cut. If, after the probe-pointed knife is introduced, Poupart's ligament is found relaxed, it should be immediately withdrawn; for, in all probability, we have to deal with one of those (fortunately exceptional) cases in which the strangulation is caused by the sac itself, and its cause is not to be sought for in the inguinal or femoral canal.

"After the ligament is cut, the hernia will almost immediately slip back if the patient is not too deeply anæstetized, or it can be returned to the abdominal cavity with the greatest ease by manipulation.

"I think it will be advisable to make the incision fully an inch from the hernial protrusion, towards the spine of the ilium; but this point should be left open until further experiences are gathered. It is quite possible that an incision of the lateral half or nearer the spine of the ilium will perhaps do as well as an incision at the median portion of Poupart's ligament nearer the strangulation.

"*The anatomical and physiological effect of cutting Poupart's ligament will be a complete relaxation of the external and internal ring and all intermediate parts of the canal, as well as of Gimbernat's ligament and the entire femoral ring.*"—*Southwestern Medical Journal.*

**Paracoto in Dysentery.**—R. G. Witherspoon, M. D., Holland's, S. C., says: E. Y., age two years, had been ill of dysentery for four days. I was called at night, June 5th. Found her in convulsions; had her placed in the warm bath, cold applied to the head and gave an enema of warm water, which was retained only a short time. Convulsions recurred about every hour, bowels moving, tormina and bloody mucous discharges every half hour. Gave calomel and ipecac, and every third hour administered rectal injections of half pint of saturated solution alum in cold water every two hours.

8 a. m., June 6th. Found her almost in state of collapse—skin cool, face pale, very restless, and constant discharge of dark green matter from bowels. Gave injection of 20 grains of ipecac in half pint of cold water, after which there was a copious discharge of green matter followed by blood, mucous, etc.

8 p. m. Found her in very much the same condition; stupor, however, had supervened, after restlessness; contracted pupils, bowels still acting and very watery. Gave fluid extract paracoto, ten drops, every four hours.

8 a. m., June 7th. Found patient playing with her dolls; warmer, and bowels under better control.

June 8th, 8 a. m. Find her still improving; convalescent. Continue the paracoto three times a day. The case progressed favorably, and at end of two weeks the child was well and hearty.

Fred. T., aged tw[illegible] years. Fell ill June 2d. I was called on the 4th and found him in very much same condition as previous patient, minus the convulsions.

He had the characteristic dysenteric discharges. Treatment was very much the same as in previous case—injections, ipecac, bismuth, opium in form of paregoric, calomel, quinine, etc. He was relieved of the urgent symptoms in a few days, but diarrhœal, and at times dysenteric discharges continued. I prescribed fl. ext. paracoto, but it could not be had before the little boy went into a condition of marasmus. He died at the end of four weeks of inanition. I believe had it been possible to have procured a reliable preparation of paracoto after the first storm of dysentery had subsided, its tonic and astringent properties would have so relieved the excessive discharges and toned up the digestive system that the result would have been a cure.—*Medical Age.*

[Paracoto, or the coto tree, native of South America.—EDITOR RECORD.]

**An Unusually Rapid Case of Graves' Disease.**—Dr. J. Mitchell Clark recently reported to the Bristol Medico-Chirurgical Society a case of Graves' disease in a girl of eighteen that proved fatal in six weeks. She came of a healthy family, commenced to menstruate at the age of fourteen, and enjoyed good health until June, 1886. During a menstrual period she then experienced palpitation of the heart, frontal headache, and pain in the præcordial region with nausea and vomiting; the next period was regular and without these symptoms. On August 4th the palpitation returned, though not so severely; but she noticed also that her eyes were becoming prominent, that her throat was larger, and that she was losing flesh without any obvious cause.

A fortnight later she was admitted into the Bristol Infirmary, when she presented all the symptoms of a typical case of Graves' disease. There was great enlargement of the thyroid, and a thrill could be felt in it; the eyes were very prominent; von Graefe's lid-sign was present; and the heart was beating very rapidly, with a forcible impulse and a blowing murmur at the base. There is no mention of sweating or of tremor At first she went on well, taking arsenic, but then vomiting and later diarrhœa set in, and she rapidly lost flesh and eventually died on September 13th, the heart's action towards the last having been extremely rapid, the emaciation great, the gums bleeding and the hair falling out. No changes were found in the nerve-centres.—*Bristol Medico-Chirurgical Journal, March, 1887.*

**Boracic Acid in the Treatment of Leucorrhœa.**—From the excellent results which are yielded by boracic acid packing in chronic suppurating otitis, Dr. N. F. Schwartz (*St. Louis Courier of Medicine*, June, 1887,) was led to employ it in a case of leucorrhœa which had resisted the most persevering use of ordinary remedies. The experiment was successful within a fortnight, and the patient has remained well for several months since. Dr. Schwartz states that he has been equally successful in a number of other cases. His manner of using it is as follows: Having first irrigated the vagina with water at as high a temperature as can well be borne by patient, a cylindrical speculum is introduced, and the vaginal walls very carefully dried, first with a soft sponge and then with absorbent cotton. This done, boracic acid in crystals is poured into the mouth of the speculum, and pushed up against the uterus and vault of the vagina with a clean cork caught in a uterine sponge-carrier, sufficient acid being used to surround and bury the intravaginal portion of cervix, filling the upper part of vagina. A tampon of absorbent cotton is then firmly pressed against the packing, and held *in situ* until the folds of the vaginal walls close over it as the speculum is withdrawn.

This should be allowed to remain three or four days, or even longer, as after this time there still remain some undissolved particles of the acid, nor will the tampon seem at all offensive. The ostium vaginæ, if examined in twenty-four hours, instead of being besmeared with the leucorrhœal secretion or discharge, presents a clean appearance, and bathed in a watery fluid, which begins to appear several hours after the packing has been placed, and in his cases this was the only discharge noticed afterwards.

However, a second or even a third repetition may be necessary, but in none of his cases, numbering nearly a score, has he found more than a second packing called for, and in many one sufficed; and in no instance has its use occasioned pain, not even inconvenience.—*Therapeutic Gazette.*

**Treatment of Pulmonary Tuberculosis by Subcutaneous Injections of Eucalyptol.**—In 1883, Roussel saw at the Exposition of Vienna a eucalyptol distilled many times, from which was separated by decantation and filtration a bitter, acrid resin, which was limpid and transparent, and possessing a finer and sweeter taste than all other essences. Roussel continued his experiments with this highly refined eucalyptol; the injections were not painful, they were easily borne, and produced no general disturbances.

After various trials, made in consumptives of all kinds and conditions, Roussel fixed upon the following treatment: During the first week he gave a daily injection of 0.20 or 0.30 cc. of eucalyptol; afterwards, three times a week, he gave an injection of 0.30 to 0.40 (from 5 to 6⅔ minims), increasing the dose to 0.80, (12 minims), which he considers it useless to pass, but it may be increased to 1 gram (15 minims) without inconvenience. The injection should always be subcutaneous, under penalty of causing great pain. Roussel prefers the hip as the place for making the injections. Three or four minutes after the eucalyptol is injected, the patients say that they detect in the mouth and nose the characteristic odor of the drug, which lasts for five or six hours during the first few days, afterwards becoming persistent.

At about the sixth or tenth injection, as a general rule, the sputa change from greenish, grayish, or reddish, heavy or viscid to white or pale yellow, and become liquid, frothy, and are easily expelled. The attacks of cough diminish in frequency and violence. The disagreeable, fetid odor which issues from the mouths of the patients ceases. The patients swallow better, they eat with more appetite, they sleep all night through, and move about more. They observe the change, and are encouraged by it. The breathing improves, and percussion does not give a dull sound in places formerly consolidated; the abnormal auscultatory sounds likewise diminish. Microscopical examination shows that the bacilli tuberculosis decrease in number after about thirty injections; after three months' treatment no bacilli are observed at all.

Roussel's experience is certainly remarkable; and his method undoubtedly demands recognition and trial on the part of other physicians.—*New Orleans Medical and Surgical Journal.*

**Directions for Vaccinating.**—G. R. C. writes to the *College and Clinical Record*: "I have repeatedly used the quill, but I have had nothing but failure; tried it on several young children, but all failed. If you recommend the quill, please send me full instructions how to use it."

The rules to be observed in vaccinating, in order to insure success, we should consider to be:

1. Secure good lymph in an active state (tube, quill, or ivory point).

2. Scrape away the horny layer of the epidermis with a thumb-lancet, until the surface becomes moist, over an area of about one-third of an inch in diameter. In primary vaccination, two such spots are prepared; usually on the left arm, at the insertion of the deltoid muscle. In girls, the outer aspect of the lower limb, just below the knee, may be preferred. "Vaccination performed over a small nævus may result in its cure.")

3. Wet the dry lymph by dipping the point in water; shake off the superfluous moi-ture, and rub the *coated* surface of the quill upon the denuded spot until the coating is deposited. If the glass tube be used, it is only necessary to break the point and apply the drop of lymph as before.

Allow the surface to dry, and prevent irritation. To accomplish this end, a small piece of waxed paper may be placed over the spot, although this is not absolutely essential.

With humanized virus, the results are very evident upon the eighth day; with pure bovine lymph, the period of incubation is several days longer—in some cases it may be several weeks. With the above method of procedure success may be expected in primary cases, and in a large percentage of re-vaccinations, *provided* the virus be good and is used while fresh.

**Treatment of Grave Epistaxis.**—M. Verneuil read a communication at the Academy of Medicine on the treatment of certain forms o' epistaxis by counter-irritation over the region of the liver. M. Verneuil began by stating that he had at first thought that the method was entirely his own, but from biographical research it turned out that he had been anticipated to a certain extent by Galen, who says that large cupping glasses applied to the hypochondria arrest nasal hemorrhage. In the first case related by M. Verneuil, the epistaxis was probably symptomatic of cirrhosis of the liver. Quinine, ergotine, and digitalis had all been tried in vain. The hemorrhage continued to recur at intervals. The second patient had suffered from nasal hemorrhage, which seemed to have been caused by the shock of a kick from a horse. In this case plugging had failed. The third was the subject of chronic nephritis, with secondary affections of the heart and liver, and the cavity of the nose had been plugged without effect, both with ergotine and perchloride of iron. M. Verneuil's treatment, which was immediately and permanently efficacious, consisted of the application over the region of the liver of a large blister.—*London Lancet.*

**Veterinary Medicine—Equine Founder.**—Horses are apt to suffer from ungual tenderness in their fore feet. The segments of the hind legs are set at such oblique angles that the hoofs do not sustain such an amount of concussion as the anterior pedals. The natural range of a horse is on a pasture, or turf, and when rode or driven over soft roads, the animal seldom gets lame from ungual inflammation or irritation—tender foot. The color of a horse has something to do with the work the fore feet will endure without development of tenderness. The sorrel and the dark chestnuts are apt to inherit a predisposition to founder; and the deep bays, with almost black legs, and black hoofs in front, rarely get lame in their fore feet. A white hoof forward is to be avoided in a purchase; it will not stand the pounding of a city's stone pavements. At the time of "shedding" in the spring, the white footed horse will be lame from ungual irritation—from pedal tenderness.

It is amusing to hear jockeys talk about such things; they will say that the horse has "corns," which must be cut by the blacksmith; then the beast will be all right—will be as good as new. In Kentucky the jockeys and experts in horse breeding will allude to a strained sinew or sprung tendon—the idea conveyed being that such lesions readily recover. They—the jockeys—don't admit that the lame horse is *foundered*, for that would hinder a sale. It is safer for them to say, that in leaping a fence or ditch the horse "sprung a tendon."

But a cure is what the owner of a foundered horse wants, and here it is:

℞. Glycerine.................................f ℥ vj,

Fowler's solution, tinct. cantharides.........*aa.* f ℥ ss.

M. S. Use on both fore feet where the hair joins the hoof, once a day for a few weeks, and a tougher kind of ungual material will grow in place of the dense and shelly stuff which has been developed under irritation. If the applications be kept up for six months; there will be almost new hoofs grown; and the horse will drive on a hard pavement without flinching. It is well to see that the "frog" is free from eczema or "thrush."

N. B.—After the founder is cured the horse should be sold, if it have acuminate ears set near together, and moved rapidly as if eager to catch a runaway sound.—*Eclectic Medical Journal.*

**Dysentery.**—Dr. Fordyce, making a digital examination of the lower rectum, in a case of this disease that was threatening badly, found a small free space above the sphincter that would hold about half an ounce. Into this *cul de sac* the fluids in the bowels above accumulated, and as soon as the space became filled, the tenesmus commenced, and an immediate evacuation followed. Above this space the whole calibre of the intestine was occluded with soft, pulpy ædematous mucous tissue, which was nearly impervious.

The treatment was rational, accordingly, successful. A soft rubber tube was carefully passed above this point through which the patient lying on his side, the colon was irrigated with 4 or 5 quarts of water as hot as could be borne. Then a quart or more of a solution of mercuric bi-chloride of the strengths of 1 to 10,000, was injected and allowed to return at once. Pain and tenesmus were at once relieved. A suppository of one grain of opium was then given, which afforded the patent the first sleep he had had since the beginning of his illness. Some pain returned after twelve hours, the treatment was repeated and the patient was cured in six days.—*Georgia Eclectic Medical Journal.*

**A young physician,** who has just established himself, and has very little practice, is noted for his braggadocio. One of the older physicians, meeting him on the street yesterday, asked him how he was coming on. "I've got more than I can attend to," was the boastful reply; "I had to get out of my bed five times last night." "Why don't you buy some insect powder?" asked the old doctor.—*Ex.*

**Lantanine; a Quinine Substitute.**—Lantanine is an alkaloid discovered by M. Negrete, and extracted from *Yerba sagrata*, of the family of verbenas. M. Buiza has observed that, like quinine, this alkaloid had some action on the circulation. It slows the nutrition, and at the same time lowers the temperature. The most delicate stomachs tolerate lantanine. Intermittent fevers that prove refractory to quinine have yielded to the influence of 2 grams of lantanine. In order to produce antipyretic effects in febrile conditions the dose employed is from 1 to 2 grams in 24 hours, given in pills of 10 cg. In intermittent fevers the drug is administered immediately after the paroxysm. Ninety-five times out of a hundred, a further paroxysm will not appear. The tincture of lantana cannot be employed, owing to its intense bitterness, which cannot be masked by syrup or wine.—*Quarterly Compendium of Medical Science.*

**Resorcin Inoculations in Phlegmon.**—Dr. Ludwig Weiss, of New York *Medical Record*, recommends very highly resorcin inoculations in phlegmon, especially of the fingers. He claims it is an effective remedy in all furuncular and phlegmonous inflammations; it abates the inflammation, if used in time, by destroying the germ locally, as well as in the lymph canals leading from the point of infection, at the same time acting as an anæsthetic on the sensitive terminal nerve filaments. Dr. Weiss makes a number of shallow parallel incisions through the integument into and around the lesion, then applies a thick layer of 10 to 30 per cent. resorcin lanoline ointment, then a piece of lint smeared with the ointment, then gutta-percha tissue, absorbent cotton, and a moist gauze bandage. The resorcin ointment must be applied abundantly and continuously. The ointment must be renewed twice daily. Relief from pain and tension ensues in from six to twelve hours.—*Quarterly Compendium of Medical Science.*

**Passage of a Large Foreign Body Through the Alimentary Canal of an Infant.**—The following case may be interesting to your readers, says Dr. C. D. Stewart (*British Medical Journal*): A child barely two months old, had, through the playfulness of an older one, got introduced into its mouth, and swallowed, a large heavy nail, fully three inches long. The parents did not call in medical assistance till they discovered the nail in a dejection. On inquiry it was elicited that during the period between the swallowing and the passing of the nail, that is to say for nine days, the child had shown no deviation from normal health. The case is interesting as showing how such a large body could pass through the primæ viæ of so young a child, without giving rise to the slightest symptoms to indicate its presence and passage.—*Quarterly Compendium of Medical Science.*

**A Specific for Diabetes.**—At a meeting of the Societe de Therapeutique, M. Martineau stated that he had been treating diabetes for the last ten years, with almost invariable success, by a method which he had borrowed from a practioner now dead.

He had hitherto made no communication on the subject, because he had wished to be perfectly certain that his conclusions were not premature. The treatment consists in the administration of a solution of carbonate of lithia and arseniate of soda in ærated water, to the exclusion of all other drinks. Besides taking this with his meals, the patient uses the same as a beverage when thirsty at other times. M. Martineau affirms that this regimen has cured sixty-seven of seventy diabetic patients he has had occasion to treat.—*London Lancet.*

**Nettle Juice.**—*Les Nouveaux Remedes* states that the best remedy for hemorrhages of all kinds is nettle juice. It is better than tannin, chloride of iron, sulphuric acid. hydrochloric acid, or ergot. The illustrious Chomel said: "This remedy is the most certain to stop hemoptysis and all hemorrhages."

The difficulty heretofore has been that the remedy was not available, because it will not keep. However, a pharmacist, M. Peneau, has conceived the happy idea of preparing a syrup from the juice. This is done at a time when the plant is in full vigor. In this form the medicine does not alter, and keeps indefinitely.

**Possible Substitutes for Cocaine in Medicine.**—Dr. A. Hughes Cennett says, that, in an investigation undertaken in 1872, he demonstrated that the physiological properties of theine, caffeine, theobromine, guaranine, and cocaine, when administered hypodermically, were to all appearances the same. "Should it be proved that they have also similar effects when applied externally to the mucous membranes, it would be of importance from an economic point of view; as cocaine is extremely expensive, while the others are comparatively cheap."

**Prolapse of Rectum.**—Dr. Hicks (in *Medical Brief*) says: The treatment of procidentia recti is simple; if a child, introduce a suppository containing from 2 to 4 grains of gallic acid, at night, washing out the rectum with cold water the morning following. Never allow the patient to sit down to stool, but remain in a semi-erect position, and never use anything to wipe the anus with, but wash with cold water, and your patient will be well in a week. If a grown person, increase the gallic acid to 4 or 6 grains.

**Atropine and Carbolic Acid.**—Professor Bartholow thinks that the physiological antagonism of atropine to carbolic acid, so valuable in poisoning by the latter, is not widely enough known. It has been proved clinically, and also experimentally in his own laboratory. The reverse antagonism does not exist. In carbolic-poisoning, atropine should be given hypodermically.—*P. S. News.*

**Spasmodic Croup in Children.**—In the treatment of laryngismus stridulus, Prof. Wallace highly approves of large doses of potassium bromide, given every hour or two; for a child two years old, he would give 6 grains every two hours. It may be given in syrup of wild cherry, or in the form of elixir of potassium bromide, which is made by the pharmacists generally.—*College and Clinical Record.*

